# Taking responsibility for health in an epistemically polluted environment

**DOI:** 10.1007/s11017-018-9444-1

**Published:** 2018-07-28

**Authors:** Neil Levy

**Affiliations:** 1 Department of Philosophy, Macquarie University, Sydney, NSW 2109 Australia; 2 Uehiro Centre for Practical Ethics, University of Oxford, Oxford, OX1 1PT UK

**Keywords:** Health care, Responsibility, Epistemology, Regulation

## Abstract

Proposals for regulating or nudging healthy choices are controversial. Opponents often argue that individuals should take responsibility for their own health, rather than be paternalistically manipulated for their own good. In this paper, I argue that people can take responsibility for their own health only if they satisfy certain epistemic conditions, but we live in an epistemic environment in which these conditions are not satisfied. Satisfying the epistemic conditions for taking responsibility, I argue, requires regulation of this environment. I describe some proposals for such regulation and show that we cannot reject all regulation in the name of individual responsibility. We must either regulate individuals’ healthy choices or regulate the epistemic environment.

Lifestyle-related factors are responsible for a huge burden of disease [1]. Many people have responded to this fact by urging regulation as a means of improving health. Such regulation might be imposed by government (say, through laws governing serving sizes) or through the actions of corporations (say, by reducing sugar in their products). Opposing such measures is a group of individuals who believe that people should make healthy choices for themselves, without handholding by paternalistic institutions. The motivations for opposition to paternalism are diverse, ranging from a libertarian antagonism toward interference in the general sphere of individual choice to worries about institutional overreach. To the extent that they form a cohesive unit, those opposed to individual regulation are unified by the conviction that people ought to take responsibility for their own health.^1^

In this paper, I argue that insofar as those on the anti-intervention side accept that individuals should take responsibility for their own healthy choices, they are committed to much more intervention than many of them would care to approve. There are epistemic conditions that must be satisfied in order for individuals to take responsibility for their choices, and satisfaction of these conditions requires the management of epistemic institutions. Therefore, those who oppose interventions on choices to improve health in the name of individual liberty, or who argue that the benefits of such interventions can be achieved at lower moral cost through personal responsibility, find themselves in an invidious position. They can oppose interventions in one area only by committing to equally far-reaching interventions in another. I hasten to add that while very many opponents of interventions face this problem, not every opponent must. Inasmuch as opponents base their positions on general libertarian principles, they face the dilemma.^2^ Those who oppose intervention on other grounds—such as those specific to particular sectors—may escape the worry. Since very many opponents of intervention are worried about regulation per se, however, the claim that they cannot escape it changes the terms of the debate significantly.

In the first section of this paper, I briefly sketch the anti-paternalistic case against interventions before turning to the positive argument that people ought to take responsibility for their choices. I show that satisfaction of this condition requires that people can reasonably be expected to guide their choices in a way that is appropriately informed—that is, that they satisfy the epistemic condition on responsibility. In the second and third sections, I present the case for thinking that people do not satisfy the epistemic condition on responsibility for their health-relevant choices, since they operate in an epistemically polluted environment. In the fourth section, I argue that bringing about ordinary people’s satisfaction of the epistemic condition on healthy choices requires far-reaching interventions into epistemic institutions, and I put forward some suggestions regarding the shape of these interventions. The fact that agents can take responsibility only if epistemic pollutants are removed from the environment, I argue, entails that those who oppose intervention face a dilemma: accept intervention into health-related choices or accept it into our epistemic institutions (or both).

## Opposition to intervention and the epistemic condition

Opposition to the regulation of health-related choices has long come from those with a libertarian or pro-market bent. It is easy to find examples on the internet of ordinary people, corporate spokespeople, and pro-market media decrying the so-called “nanny state,” while advocating personal responsibility as a solution to lifestyle disease. More interestingly, those who oppose nudges on the grounds of paternalism—surely, the most important motivator for such opposition—seem committed to holding that people ought instead to take responsibility for their choices. Paternalism is held to be unacceptable because it interferes with autonomous choice [8]. But satisfaction of the conditions for autonomous choice entails satisfaction of central conditions for moral responsibility for this choice [9]. Some people may hold, together with John Fischer [9], that autonomous choice *is* responsible choice (though not always vice versa). Others may hold that autonomy and responsibility come apart in both directions. No matter, for the purposes of my argument here: autonomous agents must satisfy the epistemic condition on moral responsibility. It is a condition of both moral responsibility and autonomy that agents be appropriately informed concerning the nature and likely consequences of their actions. This fact is enshrined in medical ethics by the requirement that patients give informed consent for medical procedures: the requirement that consent be informed is the requirement that the consenting person satisfy epistemic conditions with regard to its propositional content.

What, precisely, one must know in order to give one’s informed consent is controversial; likewise, it is controversial what the epistemic conditions on responsibility are (see [10] for discussion). While some theorists maintain that responsibility is underwritten only by what agents actually know, many others maintain that agents can be responsible for ignorant choices if they could reasonably have been expected to know certain facts about their choice.^3^ For example, an agent might be responsible for a bad diet even without knowing his food choices are bad if he ought to know this fact. Accounts which hold that agents may satisfy the epistemic condition on responsibility even when they are ignorant are less demanding than those that require actual knowledge. Accordingly, I will assume such accounts are correct.^4^ If agents cannot satisfy these less demanding conditions, they also cannot satisfy the more demanding ones, but not vice versa. Thus, by assuming the less demanding account, I make my task harder and avoid begging questions against my opponents.

Do agents satisfy these relatively undemanding conditions with regard to their health-relevant choices? There are, of course, genuine uncertainties about the links between nutrition and other aspects of lifestyle, on the one hand, and health outcomes, on the other. But satisfying the epistemic conditions on responsibility does not require certainty or even very high confidence: under a wide range of conditions, agents have been held responsible for outcomes that they justifiably believed were more likely than not to result from their actions. It seems plausible that ordinary people have a degree of justifiable confidence in at least some of the links between lifestyle and morbidity and mortality that is higher than is required for satisfaction of the epistemic condition.^5^

In the contemporary environment, we do not lack for information. On the contrary, I submit that our epistemic predicament arises not from a deficit of information but from a surplus. We are faced with a dizzying array of sources, often making conflicting claims. For instance, googling the phrase “should I vaccinate my child?” returns nearly 6000 hits. On the front page alone, hits include government-linked websites, independent journalism, forums on the parenting discussion site Mumsnet, and anti-vaccination sites like Larry Cook’s Stop Mandatory Vaccination (http://stopmandatoryvaccination.com). These sites make conflicting claims about vaccine safety—especially with regard to the purported link between vaccines and autism—about herd immunity, and about the risks of failing to vaccinate. Ordinary people lack the scientific expertise to adjudicate these claims. How is one to choose between them?

A number of philosophers have risen to the challenge of identifying criteria that ordinary people may utilize to distinguish reliable from unreliable experts [15–18]. Since assessing the credibility of sources often requires assessing the credibility of experts (e.g., scientists quoted in newspapers), I focus my discussion of ordinary people’s capacity to assess sources around the challenge of identifying credible experts. While different treatments of this topic emphasize different sets of criteria, they converge in identifying five key benchmarks for evaluating expertise: credentials, track record, argumentative capacity, agreement with consensus, and intellectual honesty. Each of these criteria is addressed in turn.

First, genuine experts have good *credentials*. They have doctoral degrees in the subject under discussion or a closely related area. They have published relevant peer-reviewed research in their field. Experts with a particularly high degree of credibility set an agenda in their discipline, as reflected by their citation count, and are honored by their peers [16]. Second, they also have good *track records*—records that consist not just in peer reviewed publications but also in a pattern of making predictions that are borne out by events. Whereas scientific expertise is esoteric knowledge, whether predictions about future events come to pass is often publicly observable and therefore exoteric knowledge [17].

Third, *argumentative capacity* consists in more than debating skill (which can dissociate from genuine expertise). Rather, genuine experts display what Alvin Goldman calls “dialectical superiority” [15 p. 95]. One expert displays dialectical superiority over another when the former expert is able to rebut the latter expert’s claims and arguments. Fourth, *intellectual honesty* is displayed by making data available to other researchers, retracting claims that have been refuted, and declaring conflicts of interest. Because people may be biased irrespective of their sincerity, one should heavily discount those experts who have a vested interest in the truth of their claims. Finally, an expert should be accorded greater credibility to the extent that her claims are accepted by a *consensus* of her peers.^6^

Philosophers who have identified these markers of expertise differ among themselves as to whether it is reasonable to expect ordinary people to be able to deploy them in order to identify genuine experts—thereby satisfying the conditions on epistemic responsibility. While all recognize that there are obstacles to utilizing these heuristics, Elizabeth Anderson [16] and Stefaan Blancke et al. [19] are explicit in claiming that ordinary people are well positioned to distinguish the epistemic wheat from the chaff.^7^ I think they are too optimistic. On very many questions—including, but not limited to, questions concerning healthy behaviors—the epistemic environment is polluted, and the extent of this pollution is sufficient to ensure that one cannot reasonably expect ordinary people to distinguish trustworthy sources from untrustworthy ones.

## Epistemic pollution

Agents can rationally choose between experts only if the criteria that distinguish genuine experts from charlatans are common sense or widely known: if agents are to satisfy the epistemic conditions on responsibility, they must know what kinds of knowledge they must utilize to guide their selection of sources (on pain of infinite regress). In fact, many, if not all, the markers of expertise identified by philosophers enjoy widespread recognition. The fact that these criteria are widely known, however, offers an opportunity to those who would use them for deception, witting or unwitting. Since expertise must be assessed through indirect markers, to mimic the markers of expertise is to mimic expertise [17]. We live in an epistemic environment that is heavily and deliberately polluted by agents who use mimicry and other methods as a means of inflating their pretense to expertise. This fact, together with the fact that such deception is widely known to occur, reduces ordinary people’s trust in expert authority and diminishes their capacity to distinguish reliable from unreliable sources.

For instance, those with an interest in deceiving the general public may set up parallel institutions that ostensibly guarantee expertise, taking advantage of the ways in which these parallel institutions mimic legitimate institutions to ensure that people are taken in. There are some egregious examples of this practice in the field of health care. For example, a small number of doctors set up the American College of Pediatricians (ACPeds) to advocate socially conservative viewpoints related to child health care. Such an organization is surely permissible, but it has had the unfortunate (and likely intended) effect of muddying debates in the public forum by misleading people into thinking that the college speaks for the pediatric profession at large. Thus, when ACPeds issued a statement condemning gender reassignment surgery in 2016 [21], many people mistook the organization’s political beliefs for the consensus view among United States pediatricians—although the peak body for pediatric workers, the American Academy of Pediatrics, has a much more positive view of gender dysphoria [22]. Insofar as the larger organization, with a broader membership base, can be expected to reflect a wider range of expert opinions and a higher degree of expertise, it is reasonable to give its views greater weight than those of the smaller organization. When ACPeds allows or encourages the impression that it speaks for the profession, it introduces an epistemic pollutant.

A yet more egregious example of such pollution involved collaborative efforts by pharmaceutical companies and the publishing giant Elsevier to produce publications mimicking peer-reviewed journals in the interest of promoting the companies’ commercial products [23]. The companies hoped to leverage the prestige of Elsevier with these fake journals to endow their promotional “research” with an air of reliability. When the deceit was uncovered, however, the effect was just the opposite: the legitimacy of the published findings was not enhanced through their publication by Elsevier, but rather the legitimacy of Elsevier’s publications—and, by extension, all academic journals—was diminished through their dissemination of deceptive and commercially interested research.

More recently, institutions of academic expertise have been subject to a large and growing outbreak of so-called predatory journals—journals that will publish almost anything for a fee. Once again, this phenomenon has the effect of making peer-reviewed journals appear less legitimate. At times, even those who work in academia may be unsure of a particular journal’s legitimacy, and there are genuine borderline cases. For example, the Frontiers contingent of journals appears legitimate—at least to me—despite the fact that authors are expected to pay a publication fee.^8^ Yet some Frontiers journals appear to have engaged in bad behavior, whether for profit or for some other motive. *Frontiers in Public Health* controversially published articles linking vaccines and autism [24] and questioning the link between HIV and AIDS [25]. Whether due to this behavior or not, Jeffrey Beall decided to add the publisher to his influential (but now sadly unavailable) list of questionable journals [26]. The controversy surrounding Beall’s decision indicates how difficult it is to make such judgments—even for professionals. If academics with expertise in relevant fields have difficulty assessing whether particular journals or particular publishers are legitimate, one cannot reasonably expect ordinary people to make such judgments. If their confidence in scientific findings is lowered across the board as the result of such epistemic pollution, one can hardly blame them.

Epistemic pollution may stem not only from counterfeit institutions of knowledge production but also from bad behavior by legitimate institutions.^9^ For example, pollution may result from attempts to game the systems put in place to track expertise. Consider institutions with a credentialing function, such as universities, bar associations, or peer review bodies. These institutions do not exist solely to credential experts. They have other functions, and these functions may come into conflict, creating pressures to inflate credentials. For example, universities have a financial incentive to inflate the expertise of their academic staff, thereby increasing their rankings, bringing in grant money, and attracting students. Systems that assess expertise can be manipulated, and many cases of such manipulation exist—take the recent example by the University of Malaysia, which attempted to boost metrics by urging its faculty to cite one another [28]. For this reason, institutions may also be slow to investigate accusations of fraud, and they may try to keep their discoveries in-house to protect their reputations.

To these sources of epistemic pollution can be added problems internal to the conduct of the scientific community, some of which have recently received widespread publicity. Consider the so-called replicability crisis. Much of the publicity to date has focused on the field of social psychology, but there is no reason to believe that this problem is confined to a single discipline. It is true that social psychology encounters some problems that do not arise in other areas (e.g., small sample sizes). But other problems in psychology are just as common, if not more common, in the field of medicine. For example, publication bias and the file drawer effect are particular and notorious problems in medicine. *Publication bias* is a distortion in what gets published, resulting from a predisposition by journals to select certain kinds of findings over others, despite the favored kind’s lacking the intrinsic scientific merit to warrant this bias. Findings might be published because they are surprising or because they concern topics that people are interested in. As Philip Kitcher notes, it is far easier to publish work on human sexual behavior than on less exciting topics [29], and standards are accordingly lower. Perhaps the single biggest source of publication bias in science is a general proclivity for positive findings. Journals are full of papers that report newfound significant correlations between variables (suggesting a causal relation between them) or show that particular interventions significantly reduce the incidence of some pathology. Some of these findings are due to chance or evaporate when some other factor is controlled for; however, publication bias means such issues often go uncorrected, since papers that show failure to replicate results are unlikely to be published.

Publication bias may affect not only what findings are published but also what research is conducted in the first place. The knowledge that failed replications struggle to get published and that, if published, their venues are unlikely to be high profile—therefore doing relatively little to advance their authors’ careers—may discourage researchers from undertaking such studies. For similar reasons, researchers may decide against further pursuing particular lines of research when initial results are negative. This practice eventuates in the *file drawer effect*—namely, the tendency for negative results to be filed away rather than submitted for publication. More pernicious still, researchers may repeat experimental protocols until the desired results are achieved. Selective publication of positive trials and suppression of negative findings may cause the efficacy of a new treatment to be overestimated—though, in reality, it may be no better, or even worse, than currently accepted treatments. Unsurprisingly, this problem is more common in industry-funded trials than in research conducted independent of industry [30].

Finally, confidence in the science that informs health-related choices in particular has been lowered by the perception that medical advice is in constant flux. First alcohol is bad for us; now it is good for us. First we should reduce fat in our diets; now fat is exonerated and sugar is the enemy. What was healthy yesterday (e.g., meat and orange juice) is unhealthy today. At least, that is the perception of many ordinary people, as science by press release dominates media coverage [31]. The apparently conflicting advice causes many ordinary people to throw up their hands. If everything is carcinogenic, why avoid anything in particular? It is intrinsically difficult to identify experts and credible claims with regard to the kinds of causally opaque systems within the purview of scientific study. This task is made even harder when ordinary people’s trust in the institutions of science is undermined.

## Identifying experts in a polluted environment

While epistemic pollution may have worsened since the publication of Goldman’s article in 2001 [15], and even since the publication of Anderson’s article in 2011 [16], both authors were already aware that ordinary people operate within a polluted environment. They, and more recently Blancke et al. [19], nevertheless maintain that experts can be identified using the criteria that they lay out. In this section, I suggest that their claims are overly optimistic. The epistemic pollution identified in the previous section makes the task of distinguishing reliable from unreliable sources too difficult to reasonably expect that ordinary people would be able to accomplish it.

The principal problem is this: markers of expertise can help to certify genuine reliability only when they themselves are not excessively polluted. However, these markers are known to be polluted, and ordinary people cannot be expected to assess whether the degree of pollution is excessive. Ordinary people know that universities do not merely certify expertise. They also aim to attract funding and manage public perceptions, and these aims may come into conflict. Ordinary people know that peer review is conducted by people with their own interests and biases. They are disposed, for instance, to see claims that vaccines are unsafe as dangerous and wild, which entails that they will have a harder time giving such claims a fair hearing. Thus, if there are individuals who present reasonable dissent from the consensus that vaccines are safe, one might expect them to have a harder time making their voices heard in ways that confer credentials. They will have trouble getting peer-reviewed publications. They will receive less funding from granting agencies—given that grants are assessed by a form of peer review, and track record in the way of peer-reviewed publications is crucial for securing funding. Universities will be less inclined to certify them as experts by conferring them with doctoral degrees or offering them faculty appointments. Blancke et al. [19] note that Michael Behe, a prominent opponent of the consensus view on evolution, is affiliated with an accredited university, but even his home institution features an official disclaimer on its departmental website [32]. Nonetheless, such a disavowal is predicted both by the view that Behe’s claims are not well supported by evidence and by the view that scientists close ranks against dissenters.

Ordinary people therefore have difficulty distinguishing genuine experts from charlatans not because they lack the capacity to identify markers of credentials but because it is reasonable to expect them to be aware that the granting of credentials is subject to extrinsic pressures. Can they do better by reference to track record, argumentative capacity, consensus, and intellectual honesty? Track record, here, consists in the making of predictions that may be verified without expertise. Unfortunately, in politically contested sciences like global warming and nutrition, the predictions made by experts can be verified only through the use of contested, highly expert knowledge. It is true, for instance, that the global warming skeptics who cite solar activity as the principal driver of climate change are committed to the claim that a decline in solar activity should mean a full cessation or dramatic decrease in warming. But it is hard for ordinary people to recognize that such skeptics are committed to this claim or to know how to assess it—especially since the same skeptics have notoriously contested global temperature records themselves. Similarly, it is difficult to identify exoteric facts about human health that can serve to falsify predictions. Health sciences use longitudinal data across hundreds of thousands of people because they deal with a causally opaque set of variables, making it difficult for experts (let alone laypeople) to identify clear successes or failures of predictions.

Argumentative capacity fares no better as a marker of expertise. The ability to rebut arguments and the manifestation of such an ability may be dissociated from one another. As many scientists who debate creationists have learned to their detriment, well-rehearsed debaters can feign dialectical superiority by having an apparent response to every objection, even if such responses are only smoke and mirrors. They can also simulate a pretense of dialectical superiority by raising so many objections and making so many points in such quick succession that their opponent is unable to rebut more than a small fraction of them—a technique known as the *Gish gallop* after a creationist specializing in this debate strategy. While dialectical superiority enables a distinction to be drawn between those who have invested much time in the consideration of a topic and those who have not, with the former more likely than the latter to have expertise, it does not allow one to discern between genuine experts and pseudo-experts who have also spent a great deal of time thinking about a particular topic.

In the debate over expertise, intellectual honesty is commonly understood in a way that risks circularity. Anderson, for instance, holds that a putative expert acts dishonestly if she does not withdraw claims that have been refuted [16]. Of course, it is the refutation itself that is contested. As a case in point, anti-vaxxers will deny that their principal claims have been disproven, maintaining that it is not they but the scientific mainstream that engages in intellectual dishonesty. Moreover, accusations of conflict of interest are often unhelpful because, as Alexander Guerrero notes [17], such conflicts typically appear on all sides. It is common for anti-vaxxers to accuse their opponents of being in the pockets of so-called Big Pharma. They may point to genuine scandals involving relations between physicians and pharmaceutical companies, such as the phenomenon of medical ghostwriting [33], in which prominent physicians or researchers put their names to papers that are largely, or even entirely, written by company representatives. The dissenting side also commonly maintains that funding from research bodies presents another pervasive conflict for mainstream scientists.^10^

Appeals to the existence of a consensus on a topic are also unhelpful, insofar as such claims are not appropriately independent of other issues. If credentialing bodies refuse to grant doctorates to dissenting researchers, for instance, then a consensus of appropriately credentialed scientists is expected regardless of whether the consensus is well supported. If data that conflict with the consensus view are suppressed—either deliberately with insidious motives or incidentally because they are difficult to publish—then the consensus has little evidential value. This point represents a generalization of Goldman’s observation that the concurrence of additional experts contributes no additional evidential weight to a claim unless such experts are sufficiently discriminating in their beliefs [15]. Although Goldman worries about excessive deference to opinion makers, other causal routes to the appearance of consensus are available. If institutions that grant credentials use inappropriate criteria for assessing expertise—such as the bare refusal to accept certain claims—then the resulting consensus is not truth conducive.

It is intrinsically difficult for ordinary people to sort through the mass of claims and counterclaims about contested issues. For each claim by a scientist, there seems to be a rebuttal by an opponent, and vice versa. While one side typically features better-credentialed experts than the other, such a pattern of distribution is exactly what one would expect if the better-credentialed side suppressed dissenting research. Such suppression might be conspiratorial, but it need not be: it could even be produced by well-meaning but biased scientists trying and failing to give their opponents a fair hearing. The claims of intellectual dishonesty that abound on both sides are largely unhelpful, since these claims of dishonesty can be adjudicated only by adjudicating the first-order issues on which they turn. Therefore, concerns about intellectual dishonesty cannot be utilized to identify reliable experts: the criteria are not sufficiently independent of one another.^11^

If one trusts the universities that credential and hire experts, the journals that publish their findings, or the institutions that fund their research, then one may utilize the many markers of expertise made available by these fixtures of academia. One can rely on them to provide evidence of a scholar’s qualifications, of her predictive failures and successes, and of good and bad behavior. But if there are questions as to whether these institutions can be trusted in the first place—if there are doubts about their integrity, concerns about the extent to which they reflect the interests of industry over patients, or notions that they may be biased (innocently or not) against dissent or blinkered in their attitudes—then the epistemic situation is much more difficult. Once trust is lost, it is difficult to restore, in part because no independent markers of reliability exist whereby public trust in the institutions that credential expertise can be calibrated.

Ordinary agents operate in what they know is an epistemically polluted environment. They know that many research findings are unreliable, that pharmaceutical companies often put profits ahead of patients, that fraud is not as rare as it should be, and (more banally) that we are all subject to biases and conflicts of interest. These problems do not exhaust those besetting the ordinary person’s ability to detect reliable science, though they are representative. Science uses sophisticated methods to detect genuine signals amid the cacophony of statistical noise. The combination of bad actors with a vested interest in distorting science (as with tobacco and climate change), institutional pressures in favor of certain kinds of findings, well-meaning but self-deceived purveyors of alternative therapies, and researchers who inadvertently use inappropriate methods has made it hard for ordinary people to separate higher-order signals—that is, signals of reliable signal detection—from all the noise that accompanies them. Given that ordinary people lack the tools that enable scientists to distinguish signal from noise, it seems unreasonable to ask them to take responsibility for their health.^12^

## Restoring trust in science

If ordinary people are to take responsibility for their own health, either they must be brought to a level of expertise sufficient for adjudicating contending claims on their own or their trust in the institutions that sanction expertise must be restored. There is little reason for optimism on the first score. Obviously, every person cannot be transformed into an expert—especially given the fact that expertise is domain specific. Everyone is a layperson in a majority of domains. It is unreasonable to count on successfully equipping the general public with critical reasoning skills and a grasp of basic statistical concepts. Moreover, the available evidence does not indicate that providing people with these skills and concepts would enable them to more reliably identify genuine experts or track truthful claims.

Teaching critical thinking skills, such as the capacity to identify argumentative fallacies, has small and short-lived benefits [36]. Moreover, short of expertise, a broader general education—even one that includes scientific schooling—seems to reap no benefits. In fact, such an education may do more harm than good: while better-educated liberal Democrats are more likely than less educated people to agree with the consensus views on climate change and evolution, better-educated conservative Republicans are *less* likely than less educated people to accept the consensus views [37].^13^ Better education and more tools for argumentation may enable those who distrust universities and institutions of science to counter their claims more effectively, exemplifying what Charles Taber and Milton Lodge [39] call the sophistication effect—that is, the phenomenon whereby more knowledge yields more ammunition (and more skills) for countering unpalatable claims.^14^

In recent decades, trust in the institutions that make and convey scientific and health claims has ebbed significantly on the political right. In fact, a general distrust in science has been on the rise among conservatives since the 1970s [42]. This distrust has generalized to universities as a whole: 58% of people who are Republican or Republican-leaning now say that colleges and universities have an overall negative effect on the United States, compared to just 19% of those who are Democratic or Democratic-leaning [43]. The same survey shows that 85% of people with Republican persuasions have a negative view of the news media [43]. Once trust is broken, claims are met with a skeptical eye because recipients cannot be confident that sources are properly independent of one another or that they filter claims according to the right kinds of properties. As seen above, better education makes Democrats more responsive to evidence produced by institutions. Presumably, this correlation exists because Democrats tend to trust these institutions. If people are to develop the ability to take responsibility for their health, then their distrust of these institutions must be addressed.

Almost axiomatically, however, restoring trust in institutions requires institutional reform. Some such reforms have indeed been proposed. For example, Guerrero [17] proposes a database of experts, which would contain records not only of their credentials but also of their predictive successes and failures. Anderson [16] proposes changes in the norms of reporting, to abolish false balance, and in online hyperlinking patterns to ensure conversation between partisans on both sides. While these changes might be salutary, I doubt that they will do much to restore trust. In fact, the knowledge that institutions are taking steps to avoid the propagation of false claims might instead serve to increase distrust among those who suspect their motives. In conjunction with, or instead of, these reforms, it is necessary to reduce epistemic pollution.

Pollution of the traditional kind is typically a collective action problem: while everyone might be better off in a cleaner world, individuals cannot make a significant difference to the broader environmental condition on their own, and those who pay the cost of local cleanup are worse off than those who do not cooperate. Collective action problems are solved by mechanisms that ensure that everyone, or almost everyone, contributes to the remedial goal. There are multiple ways of implementing such measures, but often—and especially in cases where all actors are not sympathetic to the goal of remedy—some degree of coercion is required. Epistemic pollution is also a collective action problem: while most people might be better off were their exposure to such pollutants considerably reduced, individuals cannot make a significant difference to the general epistemic condition on their own, and those who act alone are worse off than those who do not cooperate.^15^ It is, moreover, a collective action problem in which some actors do not share the remedial goal most people would like to achieve. Purveyors of predatory journals or of expensive and ineffective drugs may prefer to go on polluting, rather than have a clean epistemic environment. Thus, the solution to epistemic pollution, like that to environmental pollution, will almost certainly require some degree of coercion, whether from government or other institutions with the clout to impose penalties on those who do not cooperate.

While I lack the skills to develop detailed policy proposals, it is not difficult to imagine the kinds of steps that are needed to restore trust in institutions of knowledge. The number of predatory or fake journals must be radically reduced or else effectively contained to prevent contaminants from leaking into the scientific ecosphere. Doing so requires that legitimate open access journals be clearly distinguishable from illegitimate ones. This is a task for the scientific community as a whole. Universities should refuse to pay publication fees for journals identified as illegitimate, and researchers who publish in them should not receive credit for having done so (in the form of citations, promotions, or grant funds). Such a move would starve illegitimate and predatory journals of funds, causing most of them to close. Beall’s list was a great start to this end, but it would have been more effective had it been compiled in collaboration with a collective.^16^ As mentioned, the list was controversial. One might understand this controversy as arising from the list’s admission of what some saw as false positives. It would be better to use less demanding criteria for classifying journals as illegitimate. It would be easy for the scientific community to reach consensus on the vast majority of such journals, thus eliminating a very significant epistemic contaminant. Not only would a policy of tolerating false negatives over false positives enable the community to reach consensus, but it would also constitute a trust-building exercise with the general public insofar as a tolerance for dubious, but not obviously fraudulent, research reduces the risk of complaints by the faction of bloggers and other alternative media sources that present themselves as purveyors of skepticism about mainstream science.

Problems in the conduct of legitimate research must be addressed as well. Incentives should be put in place to encourage the replication of research—the most important incentive being a willingness to publish failed replications. Institutions might also mandate that such replications be conducted as part of their general graduate training (see [42] for a proposal along these lines). Similarly, hypotheses and methods can be preregistered to ensure that researchers do not change measures post hoc to ensure significance. Preregistration further eliminates the temptation for selective reporting of results: if all and only preregistered studies are published, one can be confident that one has the full array of data. Statistical techniques can be utilized to compensate for the file drawer effect, thereby generating more realistic effect sizes. Such techniques can also identify evidence of data manipulation, such as data dredging or *p*-hacking. These proposals are by no means novel—in fact, many are already being implemented. Prestigious journals in psychology, for example, have adopted practice standards that require bigger sample sizes, lowering the risks of chance findings, and encourage preregistration of hypotheses and methods (see [46]). If more prestigious journals follow suit, then ambitious scientists will be forced to observe these standards, thus meliorating the degree of unreliability in science.

At the same time, steps should also be taken both to reduce incentives to disseminate scientific findings by press release and to limit the extent to which mass media outlets (and, to a lesser extent, journals themselves) present research as revolutionary and earthshattering. This, too, is a collective action problem. Most researchers would likely prefer a world in which everyone refrains from hyping their research in the way that is currently all too common. Most might also prefer that media attention were not a significant determinant of prestige, promotions, and grant success. But given that media attention remains valuable to institutions and granting agencies, and since no individual researcher can change the culture by herself, researchers continue to feel compelled to play the media game in spite of themselves, which may mean representing their research as more important and revolutionary than it really is.^17^ As a result, consumers of media are left with the impression that yesterday’s findings have been overturned by today’s, and today’s findings will be overturned by tomorrow’s.

Nowhere is this more apparent than in the case of nutrition. A majority of people report that messages concerning healthy eating are contradictory and confusing, with the result that many do not place much faith in expert advice [31]. Indeed, there are significant controversies surrounding nutrition, and there have been several big changes in the nature of the advice (particularly with regard to fat). Yet a great deal has remained constant for decades. Evidence-based changes in advice are largely refinements rather than wholesale alterations—we should eat less red meat than once assumed, replace fruit juice with whole fruit, and not worry so much about eggs and butter. The advice “eat plenty of fruit and vegetables, exercise several times a week, limit alcohol, and eat fast food and snacks only occasionally” would have been endorsed by the vast majority of researchers twenty-five years ago just as it is still endorsed by researchers today. Researchers should emphasize both that their findings require replication before they are acted on and that their results should be seen to refine, rather than overturn, conventional wisdom. Reports that a certain food is associated with increased morbidity or mortality should present effect size in a comprehensible fashion, so that consumers are made aware that such risks are typically incurred only with regular consumption and, even then, remain small on the whole—after all, the doubling of a very small risk is still a very small risk. Since this is a collective action problem, those who defect from the cooperative arrangement should be appropriately sanctioned. Such sanctions might include a loss of prestige within the community of scientists, and thus diminished success at grant applications, or even official censure for those who sensationalize their findings.

Of course, the actual implementation of the agreements necessary for solving collective action problems is difficult and complex, especially with science being an international enterprise. There are at least two possible routes to effective regulation. The first is through governmental action. If the United States and the European Union ensure that funding is tied to responsible media engagement, then norms might change across the international science community, given the large proportion of scientific research funded by the countries they comprise. However, in many domains, the track record of government initiatives is not encouraging. While such regulations are often reasonable, policy often ignores expert opinion when issues are politicized. The second route is through self-regulation from within epistemic communities. One can reasonably be more optimistic about this course insofar as policymakers must remain responsive to expert opinions originating from within their official bodies. A country’s peak organizations, for instance, could regulate the conduct of science in ways that produce the same effects as government. Moreover, self-regulation by epistemic communities may also assuage worries about potential overreach, given that such communities exercise power over a much smaller domain. While, ideally, the implementation of regulation would occur in conjunction with changes in media norms, this latter goal is likely to prove overambitious in the contemporary cultural milieu, where media is fractured and cooperation unlikely. However, it may be possible to produce the same results without media cooperation: if reputable scientists withdraw cooperation with sensationalistic media, such outlets may earn a reputation for featuring only charlatans, increasing the likelihood that the public will ignore them.

None of the above-mentioned measures—some of which, again, have already been implemented (albeit patchily)—would solve the problem of distrust in science in the short term. When trust is lost, it is difficult to restore, since remedial measures taken by distrusted institutions are likely to be regarded with a jaundiced eye. In the long term, however, removing epistemic pollutants from the environment should increase trust in reliable sources of information, thereby increasing the extent to which it is reasonable to expect people to take responsibility for their health.

## Conclusion: regulation as the bump in the rug

In this paper, I have argued, first, that agents cannot reasonably be expected to take responsibility for their health in the current environment because it is epistemically polluted and, second, that the epistemic pollution is unlikely to be reparable without regulatory intervention of some kind (or perhaps multiple kinds, on the level of journals, academic societies, and government). If these two claims are correct, then regulating in one way can be avoided, thus ensuring that people can take responsibility for themselves, only by regulating in another. Regulation is the bump in the rug: it can be moved but not eliminated.

This fact is problematic for opponents of regulation broadly and for some—though not all—opponents of regulating individual choice. Whether or not it is problematic for a particular opponent depends on her specific motivations for opposing individual regulation, together with details of her views. Many libertarians (those placing a high value on the capacity to take personal responsibility) face a particularly acute problem: if they are to retain personal responsibility, they must accept regulation. For others, the problem is less acute. For example, some people may be troubled by regulations addressed to ordinary people but not by those addressed to specialists, perhaps because specialists are well poised to understand the nature and purpose of the regulations.

It is noteworthy, however, that ordinary people are among the intended beneficiaries of these regulations, which are designed, inter alia, to ensure that laypeople are exposed to research that is higher quality and more carefully reported. Some people will continue to find this approach unacceptably paternalistic. It certainly appears to conflict with the Millian insistence that since it is from the clash of ideas that truth and warrant emerge, the suppression of any opinion is wrong; and thus the regulation of epistemic pollution might plausibly be construed as a nudge to ordinary people.

I do not attempt to assess the prospects for opponents of individual-level regulation here. It is enough of an achievement to show that there is a strong case for thinking that one cannot simultaneously maintain that agents should take responsibility for their health while also opposing far-reaching regulation. If ordinary people—me and, in all likelihood, you too—are to be in a position to take such responsibility, the epistemic environment needs a cleanup.

## Footnotes

1 The claim that individuals ought to take responsibility for their own health is most explicit within writings from two very different camps: a strain of libertarianism which aims to limit governmental intervention as much as possible, and luck egalitarians, who believe that redistribution should be designed to compensate people for unchosen inequalities alone. The first kind of response is often encountered in media discussions of health issues; see [2] for discussion and representative examples. The second kind is the focus of active contemporary debate; see [3] for discussion of the range of options available to luck egalitarians. Rebecca Brown reviews the arguments against holding people responsible for their health-related choices [4].

2 As noted, the notion that agents ought to take responsibility for their own health plays a much bigger role in some varieties of libertarianism than in others. In fact, libertarian scholars within bioethics more often base their opposition to what they see as governmental overreach on accounts of the legitimate use of coercion (see, e.g., [5–7]). It may be that, unlike libertarians more prominent in the popular discourse, these libertarians can resist the dilemma I sketch. However, to the extent that their view is made more palatable by the conviction that, in the absence of regulation, individuals are effectively capable of taking responsibility for their health, the argument I advance here may decrease its attractiveness. I thank a reviewer for this journal for obliging me to clarify my thinking here.

3 Theorists who ground responsibility in akratic actions belong in the first camp. Such theorists hold that an agent is culpably ignorant only if they *knowingly* passed up an opportunity to know better. Holly Smith presents the locus classicus [11]. Rik Peels presents the most recent statement of a case for the view that agents may be responsible for their beliefs in the absence of akrasia [12].

4 For the record, I do not accept the permissive view (see [13, 14]).

5 However, the fact that there is a high degree of uncertainty in some domains might make it more difficult to satisfy the epistemic condition in those other domains in which we do possess sufficient certainty, to the extent that it takes specialist knowledge to identify those domains and those particular causal relationships about which we are justifiably confident. Ordinary people know that “the science isn’t settled” (as climate change skeptics like to say) in some domains, and this may make them more skeptical about science across the board. This introduces another possible source of epistemic pollution: highlighting genuine uncertainties in one domain may serve to undermine confidence in others. I thank a reviewer for this journal for making me think about the role of uncertainty.

6 Goldman presents an influential argument that consensus may not be a good guide for credibility because the different sources for a claim may not be sufficiently independent of one another [15]. David Coady argues that the kinds of situations in which experts show excessive deference to one another are rare [18].

7 Anderson suggests that ordinary people have the *capacity* to identify genuine experts, at least with regard to the “debate” over global warming (although it is not clear how far she would generalize this claim), but many lack the *disposition* to do so. I suspect she is mistaken about the capacity, not merely for the reasons I mention in the main text but also because she is mistaken in categorizing cultural cognition on the disposition side and not the capacity side of the ledger. The mechanisms underlying cultural cognition are a proper part of the broader mechanisms constituting judgment, not a mere input into them (see [20]).

8 Since conflicts of interest are a reason to discount expertise, it is incumbent on me to note that I have published in *Frontiers* journals on several occasions.

9 As a reviewer for this journal pointed out, these two sources of epistemic pollution may interact. On the one hand, it may be that the egregious behavior of out-and-out frauds distracts from, and thus provides cover for, those who game the system in less dramatic ways. On the other hand, involvement by academics and/or academic institutions in dubious practices may undermine their standing to criticize frauds and thus play a role in allowing these frauds to flourish. As academics come under increasing pressure to publish, they may even knowingly turn to fraudulent venues; see [27] for some recent cases.

10 One of the clearest cases of intellectual dishonesty in recent medical history is surely the Andrew Wakefield story. In 1998, Wakefield and his co-authors published a paper alleging a link between the MMR vaccine and autism. After other researchers failed to replicate his findings, Wakefield was found to have undisclosed conflicts of interest. The British General Medical Council then investigated further and found a litany of other problems, from performing unnecessary and invasive procedures on children with autism to suppressing data. The paper was retracted, and Wakefield was struck off the medical register. Those who trust the relevant institutions largely take Wakefield to be discredited and his research invalidated. But one who is disposed to distrust these institutions might see them as closing ranks against a brave truth-teller.

11 Very plausibly, the overall pattern of funding of climate skeptics and the track records of those engaged—many of whom played similar roles for the tobacco industry prior to turning their attention to climate change—provide evidence in favor of the claim that criteria of intellectual honesty should lead one to prefer one side of the debate over the other (see [34]). But it takes serious investigation to uncover these facts. Reading *Merchants of Doubt* is not sufficient, since the claim is a comparative one. Such an investment cannot reasonably be required of laypeople—not, at any rate, for every topic on which they are called to assess expert credibility. Moreover, the climate change case is a particularly egregious one: bad behavior is typically neither as overt nor as pervasive in other areas.

12 A caveat: there may be domains in which agents satisfy the epistemic conditions on responsibility; if so, the considerations advanced in this paper do not entail that it is unreasonable for agents to take responsibility in those domains. For example, Andreas Albertsen [35] argues that most people understand the actions required for oral health sufficiently well that they may be reasonably to expect to govern their behavior accordingly.

13 Better educated Republicans are also more likely to think that Obama is a secret Muslim [38].

14 A reviewer for this journal suggests, tentatively, that addressing conceptual confusions might be more helpful than the provision of more information. As far as I know, there is little data directly addressing this suggestion, but there is some reason to be pessimistic. There is evidence that even those with some college-level education in difficult concepts readily substitute more intuitive concepts for learned ones [40, 41]. Insofar as the links between lifestyle and health outcomes are unintuitive, it will likely prove difficult to address conceptual confusion effectively.

15 Jim Everett and Brian Earp note that the replication crisis is a tragedy of the commons [44]; I think it is plausible to generalize this point across a range of epistemic pollutants.

16 Beall’s list of Predatory Journals and Publishers was maintained and updated by Jeffrey Beall, an academic librarian, from 2008 to 2016. Though controversial, it was widely respected and often consulted. While it remains unclear why Beall chose to shutter the service, there is evidence that pressure from predatory publishers played a part in his decision [45]. Individuals are less able to resist such pressures than are collectives, which can share and distribute such pressures while offering mutual support. Moreover, collective decisions may be less controversial, especially if the decision-making body includes individuals with different perspectives and interests.

17 What if the research genuinely is earthshattering? I strongly suspect that rules regulating science and its reporting should not be written in ways that make explicit allowance for such eventualities. The genuinely earthshattering is sufficiently rare that regulatory bodies would do better by designing regulations that assume that the research governed by them is normal, not revolutionary, science.
